# Intestinal Atresia Leading to Intussusception: An Unconventional Submission

**DOI:** 10.7759/cureus.69723

**Published:** 2024-09-19

**Authors:** Mohammad Shahbaz Alam, Salah Eidin Elghote, Sherif Mohamed Mosad Kamel, Chanchal Kumar, Dhiraj Sidagonda Shedabale

**Affiliations:** 1 Neonatology, Zulekha Hospital, Dubai, ARE; 2 Pediatric Surgery, Zuelkha Hospital, Dubai, ARE; 3 Pediatrics and Neonatology, Zulekha Hospital, Dubai, ARE; 4 Pediatrics, Zulekha Hospital, Dubai, ARE

**Keywords:** congenital ileal atresia, intestinal atresia, intrauterine intussusception, intussusception, jejunoileal atresia

## Abstract

Intestinal atresia is often considered a sequela of the intrauterine vascular accident, frequently reported as intrauterine intussusception as the primary pathophysiology. We are reporting a case of a full-term newborn diagnosed to have ileal atresia secondary to some vascular accident that occurred late in the pregnancy leading to ileocolic intussusception.

This case will substantiate a different perspective of the previous understanding of the condition and allow the readers to further acknowledge how the type 3A variety of intestinal atresia allows the distal segment to telescope, causing intussusception. Keeping a high index of suspicion and emergency laparotomy in such conditions, with an expert surgical approach proved to play the most vital step in the survival and outstanding recovery of a delicate life.

## Introduction

Intestinal atresia (IA) may present itself anywhere along the small intestine as a solitary or multiple lesions. Jejunoileal atresia (JIA) is a type of IA that occurs in the distal part of the intestine. As a component of JIA, ileal atresia is among the common reasons for intestinal obstruction in neonates [1]. The incidence of JIA is one in 5,000 to one in 14,000 live births [2].

JIA is thought to be caused by an intrauterine vascular accident in the midgut’s mesenteric vessels, though the exact mechanism is not well understood due to the diagnosis typically being made postpartum during neonatal surgery through intraoperative assessment of bowel vascular structures [3,4]. Ischemia that occurs after the vascular event causes the fetal bowel to be reabsorbed, resulting in a blind proximal and distal bowel with a defect in the mesentery separating them. More extensive bowel defects are observed in the cases where the disruption of vascular integrity has been more proximal as compared to the distal bowel. The presence of meconium containing bile, lanugo hairs, and epithelial cells suggests that ingestion of amniotic fluid might have occurred before the vascular accident.

Though rare, in most of the literature, intrauterine intussusception (IUI) has been identified as the most probable pathophysiology behind the occurrence of IA. We report a full-term newborn referred to us on day 2 of life with progressive abdominal distension, the passage of blood mixed meconium, and bilious vomiting, diagnosed to have ileal atresia secondary to some vascular accident in the late intrauterine period leading to telescoping of the distal blind ileal segment into the ascending colon (ileocolic intussusception), that happened postnatally.

## Case presentation

A 38+6 weeks, male newborn with 3,450 grams birth weight was born vaginally to 21 years old primigravida mother, out of a non-consanguineous marriage. Referred to us on day 2 of life with the presentation of bilious-brownish vomiting, the passage of blood with meconium, and progressive abdominal distension noted since birth. Antenatally mother was booked into a private hospital during her first trimester of pregnancy. The antenatal course was unremarkable except for mild right renal pyelectasis, detected in an anomaly scan, and the occurrence of one episode of low-grade fever in the mother during labor for which she received one dose of antibiotics; however, Group-B streptococcus screen was negative. The baby cried immediately after birth, routine care was given, and the baby was shifted to the mother's side for rooming in. The baby presented with the above-mentioned symptoms after 24 hours of life, for which an x-ray and USG abdomen were done, which was suggestive of small intestinal obstruction, subsequently baby was referred to our hospital for surgical intervention and further management.

On arrival, the baby’s general condition was fair, vitals were stable. Systemic examinations revealed normal examinations of the chest, central nervous, and cardiovascular systems, however, abdominal examination showed, distension with some venous prominence, audible bowel sound, and some tenderness. Following admission baby was kept nil orally and was started on IV fluids and empirical antibiotics after sending a sepsis screen and blood culture. Urgent surgical consultation was sought, and the baby was prepared for emergency surgical laparotomy after conducting a detailed x-ray abdomen and subsequent confirmation of the diagnosis of small bowel obstruction.

**Figure 1:**
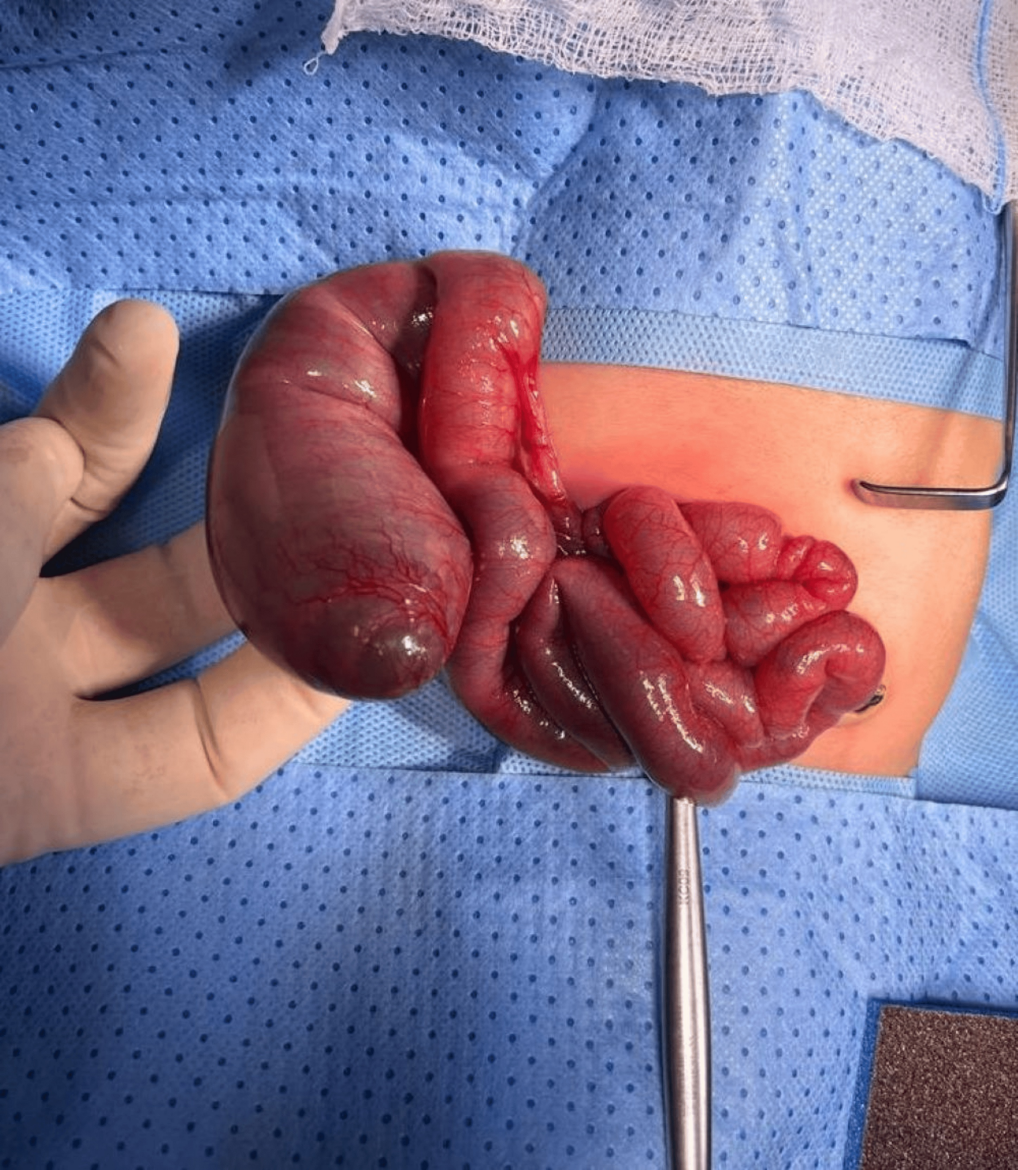
Intraoperative image showing proximal distended ileum

**Figure 2:**
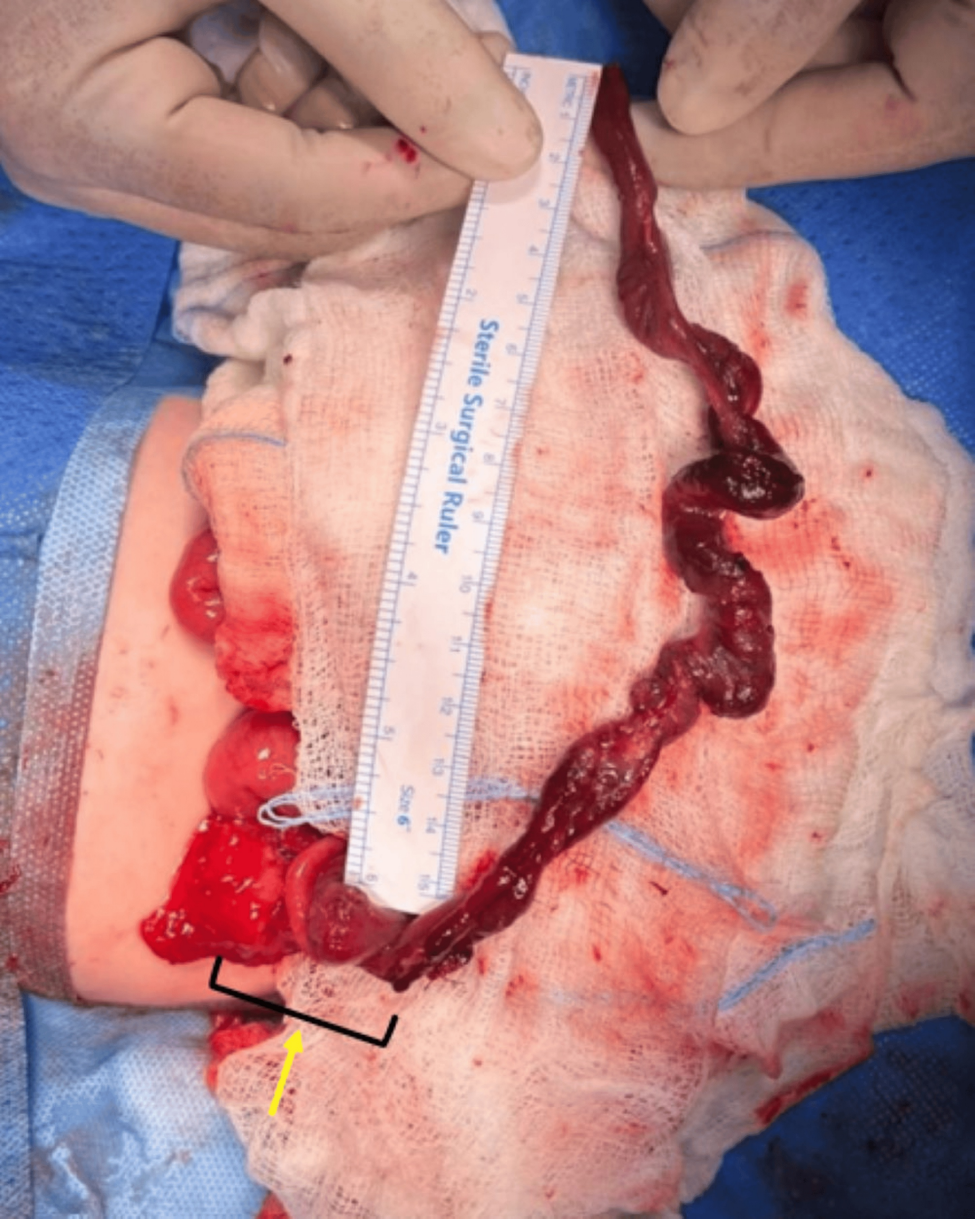
Intraoperative image of junction between distal atretic segment and intussusception

Exploratory laparotomy revealed small bowel obstruction with IA type 3A according to the Grossfeld classification. Figure 1 illustrates the proximal distended ileal segment and the bowel wall being thinned out with congestion and hemorrhage. Figure 2 depicts the 25 cm of blind distal ileum telescoped inside the transverse colon through the cecum 10 cm proximal to the ileocecal valve (ileocolic intussusception), which could be retrogradely reduced. This dead/atretic ileal segment was resected along with the cecum and appendix including the necrosed ileocecal valve. Fifteen centimeters of thinned-out proximal ileal segment was also resected, and end-to-end ileocolic anastomosis was performed. Histopathology of the resected segment revealed marked ischemia and gangrenous changes of the distal ileal segment; however, distal resected margins were viable with large bowel mucosa showing congested vessels.

The postoperative period was complicated by sepsis with coagulase-negative staphylococcus and rising C-reactive protein (CRP) which necessitated the advancement of antibiotics as per the sensitivity pattern and was given for a total of 10 days. TPN was started on day 3 and gradually weaned over 10 days. Feeds (expressed mother’s milk) were started on the fourth postoperative day and escalated to full-demand feeds as the baby tolerated well. The baby was discharged home in stable condition on exclusive breastfeeding. Follow-up visits till six months were unremarkable and presently baby is growing well with a normal developmental assessment.

## Discussion

JIA affects between one in 5,000 and one in 14,000 live births, with no preference for either sex [5-7]. While there have been reports of familial cases of JIA, the majority of occurrences are sporadic [7]. Jejunal atresia is commonly associated with anomalies such as cystic fibrosis, malrotation, congenital heart disease, Down syndrome, anorectal issues, and vertebral anomalies, whereas ileal atresia typically has fewer associated anomalies [6,8-10]. While JIA shows no association with paternal or maternal illnesses, chromosomal anomalies are present in less than 1% of affected individuals [10].

Intrauterine vascular interruption in a developing intestine in a relatively late gestational period is considered the most accepted pathophysiology behind the occurrence of IA. Volvulus, herniation, thromboembolic phenomenon involving mesenteric vessels, and vascular constriction of the bowel are some commonly known vascular disruptive events causing JIA. IUI is also a rare underlying mechanism noted only in 0.6% to 13% [11] to 25% of cases of IA [3,11].

Intussusception is the leading cause of intestinal obstruction in infants between six and 18 months but is extremely uncommon in neonates and more so in premature ones. This condition constitutes 3% of intestinal obstructions in neonates and only 0.3% (0%-2.7%) of the total intussusception cases [12,13]. While the exact etiology of intussusception is not fully understood, it is commonly linked in full-term infants to identifiable pre-existing lesions, such as duplication cysts, hamartomas, Meckel’s diverticulum, or rarely mesenchymal tumors, that act as lead points [12]. Additionally, perinatal complications like intestinal hypoperfusion, hypoxia, dysmotility, and stricture formation have been identified as potential precursors to intussusception [14]. Furthermore, intestinal hypoperfusion or ischemia causes dysmotility and stasis, but in early or recovering stages accelerated peristalsis may emerge as a functional contributing factor [12,14]. In about 8% of cases, rare anatomical lead points like diverticula, polyps, or cysts can be identified [13].

IUI as one of the causes of IA was first reported by Evans et al. [3]. Since then, several articles have been published showing IUI as a causative entity for JIA. The largest literature review of similar published cases was conducted by Chouikh et al. [15] (from 1975 to 2012), who reported 79 patients of IUI in 30 different publications. The largest study reported only two cases of IA out of a total of 24 cases of IUI [14,15].

IUI, causing JIA is seen often in full-term neonates. It usually occurs late in pregnancy, which is supported by the absence of associated anomaly, normal-sized colon, and passage of meconium in the majority of such neonates; however, only a few cases have been published in a case series of seven babies with IA [11,12,16]. At the time of surgery, the atresia process was still incomplete, indicating that the intussusception happened just before birth, as observed in our case. IUI is seldom detected by prenatal sonography after 25-30 weeks [16]; however isolated or transient ascites and dilated intestinal loops with high echogenicity depicting target-like lesions have been suggested as a few pointers toward IUI. For older infants, ultrasonography (USG) is a widely recognized and accurate diagnostic method for intussusception. Although the application of USG for diagnosing neonatal intussusception has not been extensively researched, it has proven effective in some instances [17].

In later infancy and childhood, intussusception is a frequently encountered condition and is rarely life-threatening. Diagnosing is generally straightforward when a child presents with classic symptoms, although it can be difficult in certain rare situations [14]. Recognizing the classic symptoms of intussusception is more challenging in the neonatal period, as indicators such as abdominal pain and a palpable mass are rarely present [16,17]. Moreover, prenatal intussusception presents differently clinically than it does in the neonatal period. The commonest clinical presentation observed in these cases like our index cases is rapid progressive abdominal distension and bilious vomiting within a few hours after birth. Rectal bleeding, though present in 75% [17], often occurs late in the course. Though most patients with IA pass little or no meconium [3], around 50% to 70% of IUI patients have normal meconium, like our case.

Clinical presentation and radiographs are mostly adequate to diagnose intestinal obstruction. For diagnosing neonatal obstruction, an omnipaque enema, and fluoroscopy help detect intussusception involving a colonic component [18]. When enteric intussusceptions occur without a colonic component, constituting 25%-50% of reported neonatal cases [18,19], the obstruction tends to be incomplete and the clinical signs develop slowly. Barium enema typically fails to diagnose these cases, showing a narrow or normal-sized colon instead.

Since a definitive preoperative diagnosis of intussusception is barely possible, surgery must not be delayed when there is a clear clinical impression and radiographic evidence sufficient to interpret complete intestinal obstruction [19]. Surgical treatment consists of performing a small bowel resection with end-to-end anastomosis or opting for primary intestinal derivation with delayed anastomosis. Generally, the prognosis is excellent, and follow-up assessments are usually uneventful. Nevertheless, functional obstruction may still occur post-operatively, particularly in cases of proximal IA [20].

In their literature review [15], Chouikh et al. reported that type 2 ileal atresia was identified in 14% of cases of antenatal intussusception with a complete mesentery, while type 3A, involving a mesenteric gap from mesenteric thrombosis was seen in 18% of the cases. The type of the atresia remained undetermined in 68% of cases.

The identification of a typical type 3A atresia in our case may corroborate the hypothesis of Chouikh et al. that the intussusception occurred after the atretic event [15]. The initial issue might involve successive disruptions to the intestinal blood supply, causing IA and subsequently intussusception of the distal bowel segment, although the exact reasons for this telescoping are still unclear. It could be hypothesized that defective peristalsis in the atretic segment, coupled with hypertrophied and hypercellular ganglia and increased acetylcholinesterase activity in the adjacent bowel, plays a role [20].

Histologic analysis classifies IUI into types A and B [18]. Type A features the intussuscepted bowel located at the distal blind end, where bowel structure is relatively well-preserved, probably because of the limited blood supply, according to histological studies and findings by Chouikh et al. [15]. Type B is characterized by the intussuscepted bowel being located beyond the distal blind end, where the bowel structure is necrosed and almost fully reabsorbed showing features of typical type 2 ileal atresia.

Surgeon’s perspective

Postoperative recovery in cases of IUI with IA has generally been excellent. In almost all cases where IA is identified as type 3A, intussusception occurs. This is likely due to the distal atretic segment being free, with possible hypermobility or strong bowel hypermotility leading to migration. This phenomenon is not observed in cases of type 1 or type 2 IA, where both segments remain connected after the vascular accident. Conversely, in type 3B IA, commonly referred to as apple-peel atresia, the atretic segments coil around a single axis, which typically prevents migration and the development of IUI, although it cannot be entirely ruled out. In our case, the distal segment telescoped into the transverse colon. In other type 3A cases from my experience, the intussusception was located in subhepatic, epigastric, and even left paracolic regions.

IA followed by intussusception complicates the classical ultrasound appearance of the target sign, making it challenging for radiologists to accurately diagnose the condition antenatally. As a result, diagnosis is often delayed until postnatal or even intraoperative stages.

The outcome of such cases depends on several factors, including a high index of suspicion, early diagnosis, timely surgical intervention, the timing of intussusception following atresia, and surgical expertise. In our case, the baby was born healthy but presented on the second day of life with bilious vomiting and passage of blood-stained meconium, which raised the possibility of intussusception. Although IA was not diagnosed antenatally and intussusception was not identified on postnatal USG, a prompt surgical decision was made and executed within six hours of admission. The passage of normal meconium followed by blood-stained stool, along with per-operative findings of a clean peritoneal cavity without blood, no ascites, no adhesions, and no rupture of the bowel despite significant dilatation of the proximal atretic segment, suggests that the atresia may have occurred earlier, with intussusception occurring near the end of pregnancy.

During surgery, we resected the proximal dilated atretic segment due to its unhealthy and fatigued appearance, along with the entire distal intussuscepted segment along with the necrotic ileocecal valve, and performed an anastomosis between healthy tissues. Feeds were initiated on the fourth postoperative day and escalated rapidly. The baby was discharged on the eleventh day of life. At a six-month follow-up, the baby was healthy, well-grown, and well-nourished. While the excellent prognosis could be inherent, the contributory factors discussed earlier played a vital role in the recovery. Notably, the resection of the ileocecal valve did not adversely affect the prognosis.

Limitation

The motility of the distal atretic segment leading to intussusception reaching up to the transverse colon, cannot be understood and is an interest of future avenues and research.

## Conclusions

In the literature, there are only a handful of cases that have reported IUI as a cause of IA. Thus, a careful macroscopic analysis of the distal atretic bowel segment is necessary to classify type 2 or 3A IA and gain insight into their distinct mechanism of development. In our case report, we offered a novel explanation of how IA may have developed earlier and intussusception later, just prior to delivery.

Skilled fetal USG often helps to make out the possibility of IA and or intussusception causing intestinal obstruction. Most of the reported cases revealed an excellent prognosis as in our case. However, a lack of proficient continuation of care from prenatal diagnosis to post-operative care can make the situation fatal. Hence, a high index of suspicion of intestinal obstruction, and in-utero maternal transfer to higher centers with the availability of equipped pediatric surgery could make a difference in the survival of such neonates. Aggressive pre-operative stabilization, immediate post-partum emergency laparotomy, and post-operative care by skilled neonatal teams proficient in handling such cases can remarkably improve the outcome. Our report also highlights, that removing an unhealthy ileocecal valve does not negatively affect the prognosis and might be safely undertaken in particular scenarios.
